# Biomimetic Cellulose Nanocrystals Composite Hydrogels: Recent Progress in Surface Modification and Smart Soft Actuator Applications

**DOI:** 10.3390/nano15130996

**Published:** 2025-06-26

**Authors:** Yuzhu Cui, Zekai Wang, Mingliang Zhao, Zhihui Wang, Lu Zong

**Affiliations:** Key Laboratory of Rubber-Plastics, Ministry of Education/Shandong Provincial Key Laboratory of Rubber-Plastics, Qingdao University of Science & Technology, Qingdao 266042, China; 13773903707@163.com (Y.C.); 18463161473@163.com (Z.W.); z15621493157@163.com (M.Z.); 19862212767@163.com (Z.W.)

**Keywords:** cellulose nanocrystals, hydrogels, biomimetic, surface modification, soft actuator, mechanical properties

## Abstract

Cellulose nanocrystals (CNCs), derived from renewable biomass, have emerged as a pivotal component in the design of biomimetic composite hydrogels due to their exceptional mechanical strength, biocompatibility, and tunable surface chemistry. This review comprehensively explores recent advancements in surface modification strategies for CNCs (physical adsorption, chemical grafting, and bio-functionalization) and their impacts on the structure and properties of hydrogel networks, with particular emphasis on mechanical properties. Future applications in light/thermal/electrical-responsive soft actuators are critically analyzed. Guided by biomimetic design principles, the anisotropic mechanical responses induced by CNC-oriented alignment are explored, along with their cutting-edge advancements in soft robotics, wearable sensing, and biomedical devices. Perspectives are provided on future directions, including multi-stimuli synergistic actuation systems and sensing-actuation integration architectures. This work establishes a fundamental framework for designing CNC-enhanced smart hydrogels with tailored functionalities and hierarchical structures.

## 1. Introduction

In recent years, the rapid development of flexible electronics, soft robotics, and biomedical engineering has driven the urgent need for new smart materials [1,2]. Smart hydrogel actuators, as the core materials in this field, have developed into the focus of interdisciplinary innovative research due to their unique bionic response mechanism and tissue compatibility. In the field of industrial precision manufacturing, hydrogel-driven micro-grippers can effectively reduce damage rates of precision components (≥40%) and lower scrap costs (750 million/year). It is predicted that the market size of industrial-grade smart hydrogels will exceed 30 billion by 2030. As a class of highly water-containing materials composed of three-dimensional cross-linked networks, hydrogels not only perfectly replicate the flexibility and ductility of biological tissues, but also realize the dynamic response to multimodal external stimuli, such as temperature, pH, light, electric field, etc., through chemical modification and composite technology, so as to show adaptive capability comparable to that of living organisms in complex environments [3,4,5]. Hydrogel actuators have attracted much attention as smart soft-body materials in the field of bionic robotics, but their practical applications are still limited by the combined challenges of mechanical properties and dynamic responses [6]. Traditional poly-N-isopropylacrylamide (PNIPAm)-based hydrogel actuators are widely used in the field of temperature-controlled actuation, but they exhibit significant performance deficiencies: their tensile strengths are generally lower than 100 kPa, and their compressive modulus is less than 50 kPa, making it difficult to withstand repetitive mechanical loads; the swelling kinetics are limited by thermal stimuli, and the response time of the phase change is up to tens of minutes; and there are environmental sensitivities leading to the phenomenon of dehydration and embrittlement due to high environmental sensitivity, which is prone to structural failure in humidity fluctuation environments. More critically, the realization of multi-physical field coupling often requires complex chemical modification, and the synergistic control of different stimulus-response mechanisms has become a technical bottleneck.

**Figure 1:**
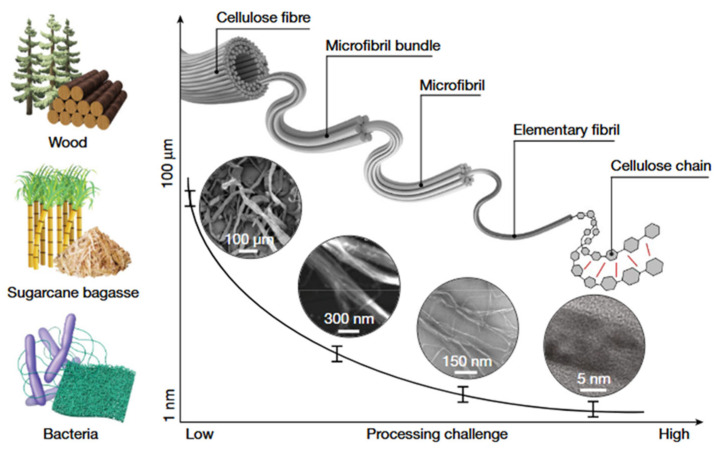
The source of cellulose and the nanosizing process [7]. Copyright 2021, Springer Nature.

**Figure 2:**
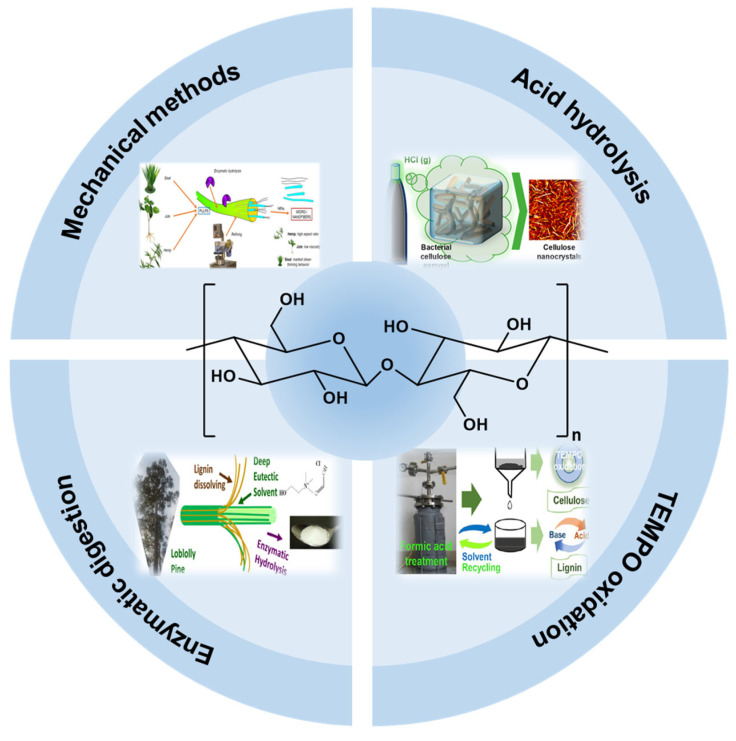
The preparation methods of CNCs, including mechanical methods [18] (Copyright 2022, Multidisciplinary Digital Publishing Institute, Basel, Switzerland), acid hydrolysis [19] (Copyright 2019, American Chemical Society, Washington, DC, USA), TEMPO oxidation [20] (Copyright 2019, American Chemical Society), and enzymatic digestion [21] (Copyright 2017, Elsevier Ltd., Amsterdam, The Netherlands). Reproduced with permission.

To address the above issues, researchers have achieved a breakthrough in material performance by introducing cellulose nanocrystals (CNCs) to build a bionic composite system. CNCs are nanomaterials derived from natural cellulose with high aspect ratios and excellent properties, usually presenting a rod-like structure with a width of 3–50 nm and a length of several micrometers, as in Figure 1 [7,8]. CNCs have a high degree of crystallinity, typically between 49% and 95%, depending on their source and preparation method. CNCs are mainly derived from cellulose fibers in plant cell walls, and their preparation methods mainly include acid hydrolysis [9], TEMPO oxidation [10], enzymatic digestion [11], and mechanical methods [12], as shown in Figure 2 and in a detailed comparison in Table 1. Mechanical methods are used to break down cellulose raw materials into nanoscale crystals by methods such as mechanical milling or high-pressure homogenization, which makes it easy to obtain cellulose nanofibers (CNFs) rather than CNCs alone, and usually needs to be used in conjunction with chemical/biological methods. The fullest and most widely used protocol studied for producing CNCs is the hydrolysis and esterification of cellulose with concentrated sulfuric acid in less than 2 h. Sulfuric acid is a strong acid, and high concentrations of protons can attack glycosidic bonds more rapidly in the ordered regions of cellulose. As these bonds break, the cellulose chains become shorter and the average degree of polymerization decreases until the disordered regions are completely degraded and a leveling off degree of polymerization (LODP) is reached [13]. If the hydrolysis reaction is allowed to continue, the polymerization degree and CNCs length of the cellulose chains will continue to decrease, but very slowly compared to the initial decrease. Typically, when the LODP is reached, the acid and cellulose slurry are quenched to terminate the reaction. At this stage, sulfated CNCs can be easily centrifuged into pellets because the high ionic strength of the quenched slurry shields them from electrostatic repulsion, and the acid is subsequently removed by centrifugation and dialysis. Finally, the aqueous CNC suspension is sonicated with an ultrasonic probe and filtered to remove any unhydrolyzed cellulose or impurities. In addition to sulfuric acid, inorganic acids such as hydrochloric acid and phosphoric acid, and organic acids such as oxalic acid and formic acid can also be used to hydrolyze cellulose for the production of CNCs [14,15,16]. TEMPO oxidation is a highly efficient and selective oxidation of cellulose, which is commonly used to prepare surface carboxylated CNCs [17]. Under TEMPO catalysis, the primary hydroxyl group (-CH_2_OH) at the C6 position of the cellulose glucose unit is oxidized to carboxyl group (-COOH), and a small amount of keto group (-C=O) is generated, which disrupts the hydrogen-bonding network of cellulose, and the nanocrystals are isolated in conjunction with the mechanical treatment. Unfortunately, the high cost, toxicity, and long reaction time of the TEMPO oxidation method for the preparation of CNCs limit the scale-up of this method.

**Table 1:** Detailed comparison of CNC preparation methods.

Preparation Method	Core Process	Dimensions of CNCs	Applications	Advantages	Disadvantages	Ref.
Sulfuric acid hydrolysis	64% H_2_SO_4_, 45 °C, 45–90 min	Length 100–200 nm, diameter 5–20 nm	Reinforced Composites (Hydrogels/Plastics), Pickering emulsion stabilizer	Mature process, high yield (~70%), sulfate ester groups improve dispersibility	Low thermal stability (sulfate decomposition), extensive washing required, equipment corrosion	[19,22]
Hydrochloric acid hydrolysis	6 reflux boil (105 °C), 2–4 h	Low (~14–28 nm)		High thermal stability, no charged groups	Aggregation-prone, poor dispersibility	[23]
Phosphoric acid hydrolysis	85% H_3_PO_4_, 50 °C, 120 min	Moderate (~11 nm)		High thermal stability, excellent dispersibility, suitable for biomaterials	Harsh conditions, larger dimensions	[14,24]
Oxalic acid hydrolysis	50% (COOH)_2_, 90 °C, 6 h ultrasonic	Uniform dimensions, tunable aspect ratio		Eco-friendly, high yield (>80%), high thermal stability	Poor dispersibility	[16]
Enzymatic Hydrolysis	Cellulase mildly hydrolyzes high-pressure homogenization	High (~28–50 nm)	Tissue engineering scaffolds, flexible sensor substrates	Mild conditions, eco-friendly, high selectivity	Low efficiency, time-consuming, high cost	[21]
TEMPO Oxidation	TEMPO/NaClO_2_ oxidation ultrasonic crushing	Very high (>150, nanofibers)	Biomedical vectors (Targeted Delivery), conductive hydrogel electrodes	High carboxyl content (easy functionalization), excellent dispersibility	Expensive oxidant, over-oxidation/chain scission risk	[20]
Mechanical methods	High-pressure homogenization/ball mill, deep delignification pretreatment	Low crystallinity (40–60%), wide size distribution (50–1000 nm)	Rheology Modifiers (Coatings/Food), Aerogel insulation	Green and environmentally friendly, process is safe and non-toxic	Poor uniformity, poor stability, and low efficiency	[18]

**Figure 3:**
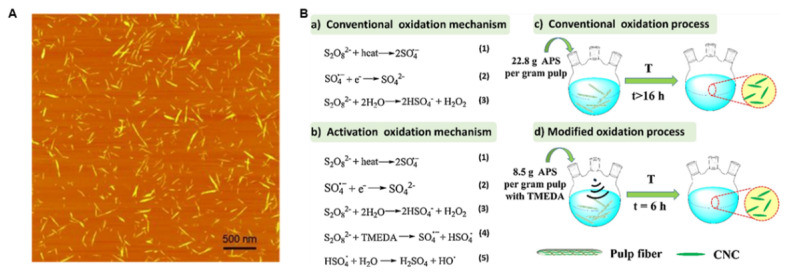
(**A**) Height mode AFM images of CNCs from flax (5 μm) [25]. Copyright 2011, WILEY-VCH GmbH (Weinheim, Germany). (**B**) Illustration of the conventional and modified APS oxidation mechanism and oxidation process [26]. Copyright 2020, Elsevier Ltd.

Additionally, the latest research indicates that the APS oxidation method for producing CNCs can eliminate certain non-cellulosic plant components during the preparation process, enabling one-step CNC production without the need for pretreatment. In 2011, Leung first proposed the use of ammonium persulfate (APS) to prepare CNCs in Figure 3A. Compared to other oxidants, APS offers advantages such as strong oxidative capacity, low toxicity, cost-effectiveness, and high water solubility [25]. However, this method requires a large amount of APS and has a long reaction time, which prevents large-scale production. Liu et al. [26] proposed the use of N, N, N’, N’-tetramethyl-ethylene-diamine (TMEDA) as a redox initiator, which can be used to form a redox initiation system with APS and act as an electron donor to catalyze the decomposition of S_2_O_8_^2−^, generating sulfate radicals, hydroxyl radicals, and hydroxyl sulfate radicals, in Figure 3B. The introduction of an ultrasonic pulverization step in the modified oxidation process can reduce the length of the fibers while increasing the surface area and improving the preparation efficiency.

## 2. CNCs Surface Modification Strategies and Their Effects on Hydrogel Properties

### 2.1. Surface Modification of CNC

Due to the large number of hydroxyl groups on the surface of cellulose nanocrystals, these polar groups cause them to strongly aggregate in water through hydrogen bonding, forming clusters that are difficult to disperse. Such agglomerated CNCs cannot be homogeneously dispersed in the polymer network, and surface modification of cellulose nanocrystals can control their dispersion, interfacial, and self-assembly properties, as well as endow the materials with new design functions. Meanwhile, the large number of hydroxyl groups makes CNCs highly hydrophilic but poorly compatible with nonpolar polymer matrices, which can be chemically modified to introduce hydrophobic groups, such as carboxyl groups and amine groups, to reduce the surface energy and improve the compatibility with hydrophobic polymers. CNCs’ surface modification strategies, shown in Table 2, can be categorized into three principal approaches: physical modification, chemical modification, and bio-functionalization. Each strategy targets specific interfacial engineering goals to optimize CNC-polymer synergy in composite hydrogels.

Physical adsorption modification is the adsorption of modifiers onto the CNCs surface by physical forces (e.g., van der Waals forces, electrostatic forces, etc.) [27]. To date, most CNC hydrogel studies have focused on the direct physical incorporation of CNCs as fillers/enhancers into the polymer hydrogel network. Common preparation methods include simple homogenization and physical entanglement of the polymer network, free-radical polymerization of polymer species in CNC suspensions, UV/ion-mediated cross-linking of the polymer network around the CNCs, and cyclic freeze–thaw processing [28]. CNCs are usually physically incorporated into the hydrogel at low concentrations, with the majority of studies employing concentrations in the range of 0.1–5 wt%; this incorporation results in an increase in the hydrogel’s shear storage modulus by a factor of 35 and uptake in water up to 1500 times its dry weight.

**Figure 4:**
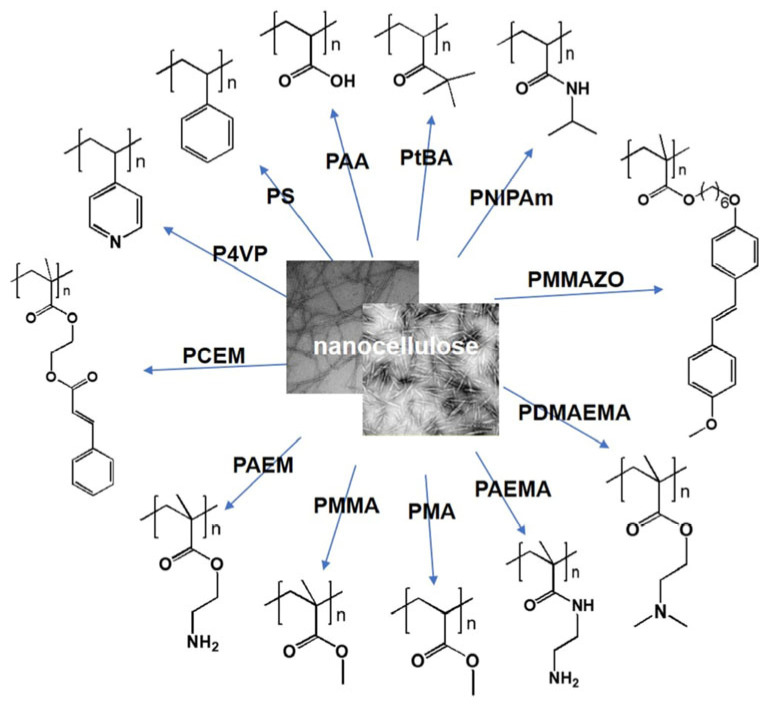
Polymers grafting from nanocellulose via surface-initiated atom transfer radical polymerization [38]. Copyright 2021, WILEY-VCH GmbH.

Since physical modification of CNCs relies solely on hydrogen bonds, electrostatic interactions, or van der Waals forces, these forces are prone to disruption under swelling or mechanical stress, leading to the detachment of CNCs from the network and a reduction in the long-term stability of hydrogels. Considering long-term stability and biocompatibility, chemically grafted CNCs are predominantly adopted in hydrogels. Chemical modification involves the introduction of new functional groups onto CNCs’ surfaces through chemical reactions. This approach enables precise incorporation of functional moieties (e.g., fluorescent labels, responsive groups), thereby endowing hydrogels with intelligent responsiveness (e.g., pH/temperature sensitivity, light-controlled drug release) [29,30,31]. A more robust modified layer is achieved, enhancing the compatibility and interfacial interactions between CNCs and the matrix. Additionally, chemical modification improves the dispersibility of CNCs in hydrogels, preventing network defects caused by aggregation. For instance, CNCs oxidized by TEMPO were reported by Rana et al. [32], where the negatively charged surface facilitated stable dispersion via electrostatic repulsion, allowing uniform embedding into alginate hydrogels. Chemical grafting modifications include: (1) Esterification reaction: Acid anhydrides (e.g., acetic anhydride, maleic anhydride) or acyl chlorides are utilized to form stable urethane bonds through p-maleimidophenyl isocyanate cross-linker molecules, and then react with hydroxyl groups of CNCs to form hydrophobic ester bonds [33,34]; (2) Etherification: Alkylating agents (e.g., ethylene oxide, chloroacetic acid) are employed to react with hydroxyl groups, introducing functional groups such as carboxymethyl or hydroxypropyl [35]. Carboxymethylated CNCs, characterized by negative charges and high aqueous dispersibility, are widely used to construct pH-responsive hydrogels for intestinal-targeted drug release [36]; (3) Oxidation: TEMPO-mediated oxidation is applied to introduce carboxyl groups onto CNCs; (4) Silane modification: Silane coupling agents (e.g., 3-(2-aminoethylamino) propyldimethoxymethylsilane (AEAPMDS), Vinyltrimethoxysilane (VTMS)) are used to graft siloxane chains onto CNC surfaces, forming hydrophobic protective layers. For example, Zhu et al. [37] coupled AEAPMDS to nanocrystals by first hydrolyzing the silane coupling agent into silanol, which then underwent condensation with hydroxyl groups on the CNCs. Separately, CNC hydrogels with varying mass fractions (1 to 3 wt%) were prepared via ultrasonication for 5 min in an ice bath. In addition to chemical graft modification of small molecules, chemical covalent grafting of polymers (Figure 4) is frequently used with a wide variety of functional polymers.

**Figure 5:**
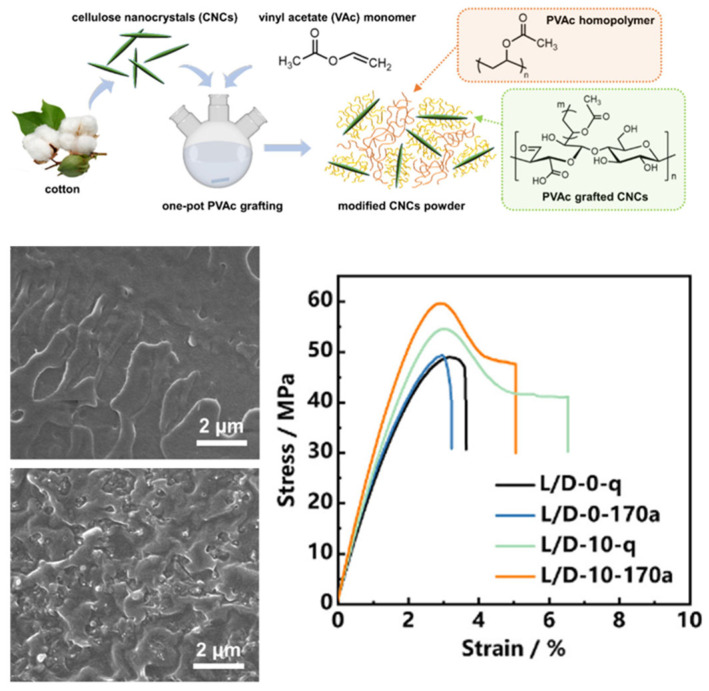
Schematic illustration showing the preparation process of PVAc-modified CNCs, the cryo-fractured surface of L/D-0 and L/D-10, and stress–strain curves of quenched (represented by the letter “q”) and annealed (represented by the letter “a”) L/D-0 and L/D-10 [39]. Copyright 2025, Elsevier Ltd.

In another study by Wu et al. [39], poly(vinyl acetate) (PVAc) chains were grafted onto CNC surfaces via efficient free-radical polymerization in an aqueous medium, rendering CNCs compatible with conventional melt-processing techniques [40]. It was demonstrated that the dispersion state of CNCs in a polylactic acid (PLA) matrix and the interfacial interactions between PLA and CNCs could be tailored by adjusting the PVAc grafting density. Further investigations revealed that well-dispersed CNCs actively contributed to the reinforcement of PLA (as indicated in Figure 5). These findings elucidate the nucleation and reinforcement mechanisms of polymer-grafted CNCs, providing theoretical guidance for the industrialization of high-performance bio-based nanocomposites.

Biological modification of CNCs refers to the precise regulation of their surface chemistry and functionality through biological methods or the directed integration of bioactive molecules [41,42,43]. This approach overcomes the performance limitations of natural cellulose materials and endows them with high-value functional characteristics. For example, enzymatic reactions (e.g., cellulase-mediated directional cleavage, oxidase-mediated introduction of active groups) or microbial-assisted synthesis (e.g., bacterial cellulose co-assembly) can be employed to reconstruct the surface chemical microenvironment of CNCs at the nanoscale [44,45]. Alternatively, bioactive molecules such as peptides, polysaccharides (e.g., chitosan, hyaluronic acid), and natural antibacterial components (e.g., plant polyphenols, lysozyme) can be integrated via covalent coupling or physical encapsulation, constructing hierarchically ordered biomimetic interfaces [46,47,48].

**Table 2:** Surface modification strategies for cellulose nanocrystals (CNCs), categorized into covalent and non-covalent methods.

Modification Type	Modification Method	Method Description	Applications	Advantages	Disadvantages	Performance Impact	Ref.
**Physical Modification**							
Surfactant Adsorption	Cationic (CTAB), nonionic (PEG) adsorption via electrostatic/hydrophobic interactions	Improves hydrophobicity; enhances polymer compatibility	Drug carriers (hydrophobic drugs); composite compatibilizers	Simple operation, reversible; preserves CNC crystal structure	Weak bonding (easy desorption); poor thermal stability	Dispersibility significant improvement; interfacial compatibility improvement; thermal stability reduction	[49,50]
Plasma Treatment	O_2_, N_2_ plasma treatment	Introduces polar groups (-COOH, -OH); increases surface roughness	Enhanced composite interfaces; biomedical scaffolds	Eco-friendly (solvent-free); uniform surface modification	High equipment costs; over-treatment may damage CNC structure	Surface activity improvement; interfacial adhesion improvement; thermal stability no change	[51,52]
Ultrasound-Assisted Dispersion	High-energy ultrasound deagglomeration	Improves CNC dispersion in solvents/matrices	Nanocomposite dispersion; Pickering emulsion stabilizers	Rapid and efficient; preserves chemical integrity	Prolonged ultrasound reduces aspect ratio; high energy consumption	Dispersibility significant improvement; aspect ratio reduction; crystallinity no change	[11]
**Chemical Modification**							
Esterification	Reaction with anhydrides (acetic/EDTA dianhydride) or acyl chlorides	Introduces hydrophobic chains or carboxyl groups	Hydrophobic composites; fluorescent material carriers; flame retardants	Controllable DS (0–63%); dramatically improves organic solvent dispersion	Strong acids/high temperatures may reduce crystallinity; organic solvent pollution	Hydrophobicity improvement; thermal stability improvement; functional sites	[53]
Silanization	Hydrolysis-condensation of alkoxysilanes (APTES, GPTMS)	Introduces alkyl chains or epoxy groups	Rubber/epoxy reinforcement; sensors	Enhanced thermal stability; provides reactive functional groups	Multi-step reaction; solvent exchange challenges	Thermal stability: Si-O bonds resist high temperatures (>300 °C); interfacial strength improvement; Dispersibility improvement organic compatibility	[54,55]
TEMPO Oxidation	NaClO/NaBr/TEMPO system oxidizes C6-OH to -COOH	High-density carboxylation (>1.5 mmol/g)	Metal ion carriers; fluorescent material templates	Mild reaction (pH 10); carboxyl groups enable further functionalization	May cause cellulose chain scission; expensive reagents	Colloidal stability improved; Reactivity: carboxyl supports amidation/esterification; crystallinity (5–10%)	[56]
Polymer Grafting	**Grafting to**: Pre-synthesized polymers (PCL, PEG) coupled to CNCs; **Grafting from**: ATRP/RAFT polymerization initiated from CNC surface	Core-shell structures or interpenetrating networks	High-toughness composites; stimuli-responsive gels	High grafting density (“grafting from”); controllable molecular weight	Steric hindrance limits “grafting onto”; complex initiator modification required for “grafting from”	Toughness improved; thermal stability: Polymer layers protect CNCs; dispersibility well	[57,58]
**Biological Modification**							
Enzymatic Hydrolysis	Cellulase selectively hydrolyzes amorphous regions	Green CNC preparation	Food packaging; biocompatible materials	Eco-friendly (aqueous phase); mild conditions	Low yield (<30%); slow reaction (>48 h)	Crystallinity > 80%; Size uniformity improvement; thermal stability: T_max_ > 300 °C	[59]
Enzyme-Assisted Assembly	Laccase/peroxidase modifies surface groups	Enhances interfacial bonding or functionality	Biosensors; tissue engineering scaffolds	High selectivity; excellent biocompatibility	High enzyme cost; complex process	Bioactivity; interfacial bonding improvement	[53]

### 2.2. Effect of CNC on Hydrogel Properties

Hydrogels are three-dimensional networks formed by cross-linked hydrophilic polymers. Their high-water content (exceeding 90%) endows them with biomimetic softness and extensibility resembling biological tissues. However, pure polymeric hydrogels generally suffer from insufficient mechanical properties (e.g., tensile strength < 100 kPa) and poor environmental stability [42]. The incorporation of CNCs as reinforcing phases addresses these limitations by forming scaffold-like structures within the hydrogel network. Through hydrogen bonding, electrostatic interactions, and other interfacial forces, CNCs establish robust adhesion with the polymer matrix, significantly enhancing the tensile strength, toughness, and viscoelastic modulus of hydrogels [60,61]. This enhancement provides opportunities for applications in biomedical, pharmaceutical, and industrial fields. The key to achieving effective reinforcement lies in the homogeneous dispersion of nanocrystals within the hydrogel matrix. Modifying the surface chemistry of CNCs improves their interfacial compatibility and interactions with hydrogel polymers, thereby optimizing dispersion stability, interfacial adhesion and mechanical properties.

Examples of the use of CNC alone in hydrogels are rare, due to the fact that CNC tends to agglomerate in the polymer network, leading to the presence of stress concentration points within the hydrogel, which reduces the mechanical strength and triggers crack extension. Surface modification of nanocellulose often needs to be adapted to the specific application.

#### 2.2.1. Physical Blending for Improved Mechanical Properties

Owing to their high specific strength, modulus, and aspect ratio, CNCs are well-suited as low-content reinforcing agents in polymer matrices. The reinforcement efficiency of these nanoparticles primarily arises from their high specific surface area and the formation of a rigid percolating network within the polymer matrix. However, dispersing CNCs in non-polar environments presents significant challenges, typically necessitating surface modification to enhance interactions with the surrounding matrix and potentially impart additional functionalities to the composites. Some studies reported to date indicate that the ATRP method yields promising results. This technique achieves excellent compatibility between the nanocellulose filler and the matrix, consequently yielding composites with superior tensile strength compared to neat polymer.

Bai et al. [62] successfully modified CNC surfaces via metal-free photoinduced electron transfer atom transfer radical polymerization (PET-ATRP). Self-healing hydrogels possessing outstanding toughness and mechanical strength are particularly crucial for practical applications. The prepared P4VP-CNC hybrid material (CNCs@P4VP) serves as a green reinforcing agent, enabling the fabrication of self-healing nanocomposite hydrogels through electrostatic interactions. Consequently, poly(4-vinylpyridine) (P4VP)-grafted CNCs proved to be an excellent filler for producing self-healing poly(acrylic acid) (PAA) hydrogels with robust mechanical strength. The nanocomposite hydrogels exhibited exceptional mechanical properties (6.6 MPa at 921.6% strain) and self-healing capability (85.9% recovery after 6 h). This study provides a viable and green approach for designing novel high-strength self-healing nanocomposite hydrogels.

**Figure 6:**
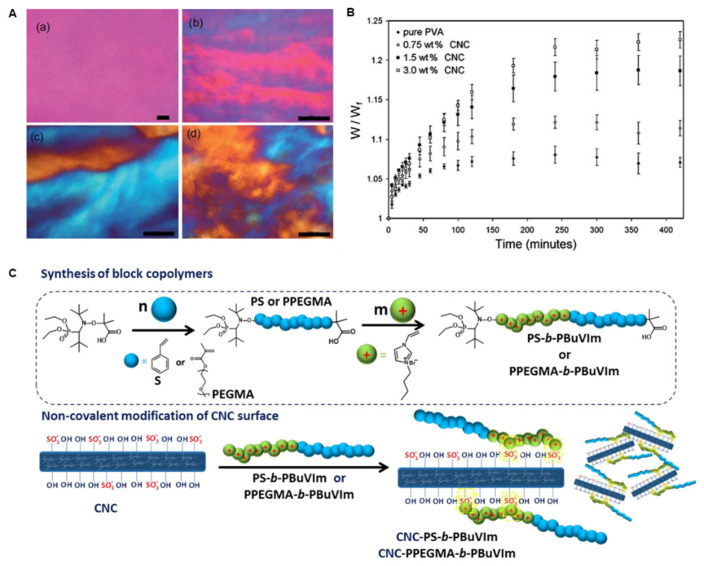
(**A**) Polarized optical micrographs of hydrogel samples after five freeze-thaw cycles: (**a**) pure PVA, (**b**) 0.75 wt% CNC, (**c**) 1.5 wt% CNC, and (**d**) 3.0 wt% CNC. Scale bars are 100 mm for (**a**), and 500 mm for (**b**–**d**). (**B**) Swelling ratio, *W*/*Wf*, plotted against time for hydrogel samples with varying CNC contents. (**C**) Synthesis of PPEGMA-*b*-PBuVIm and PS-*b*-PBuVIm and subsequent non-covalent functionalization of CNC. Copyright 2011, Royal Society of Chemistry. (**A**,**B**) [28] Copyright 2022, Wiley-VCH Verlag [63].

Abitbol et al. [28] incorporated CNCs into poly(vinyl alcohol) (PVA) hydrogels prepared by repeated freeze–thaw treatment. The CNCs-added hydrogels exhibited better structural stability and unique microstructures characterized by ordered structural domains of CNCs. Due to the hydrophilicity of cellulose and the decrease of PVA crystallinity, the water absorption of the hydrogels increased with the increase of CNCs content. Upon applying uniaxial confined compression, the CNCs-loaded samples showed an enhancement effect, and the elastic modulus of the PVA-CNC samples increased with respect to the pure PVA hydrogel (Figure 6A). Hydraulic permeability values were derived from stress transients: at strains of 15% to 20% or greater, the permeability of all samples approached a plateau value, reflecting the phenomenon of impeded flow in soft gels that have been compressed, densified, and dehydrated (Figure 6B).

Torres–Rocha et al. [63] used block copolymers to functionalize CNCs surfaces, in which a cationic block was anchored to the anionic CNCs surface by complexation, and another block acted as a stabilizing block to provide dispersibility in various solvents. In Figure 6C, the polymerization of the block copolymers poly(poly(ethylene glycol methacrylate)-b-poly(N-butyl-N’-vinylimidazolium bromide) (PPEGMA-b-PBuVIm) and poly(styrene)-b-PBuVIm (PS-b-PBuVIm) was first synthesized by nitrogen oxides mediated polymerization of the block copolymers poly(poly(ethylene glycol methacrylate)-b-poly(N-butyl-N’-vinylimidazolium bromide) (PPEGMA-b-PBuVIm) and poly(styrene)-b-PBuVIm (PS-b-PBuVIm), which was then noncovalently adsorbed on the surface of the CNCs and modified CNCs in organic solvents to produce stable dispersion.

#### 2.2.2. Chemical Grafting for Hydrogel Functionalization

**Figure 7:**
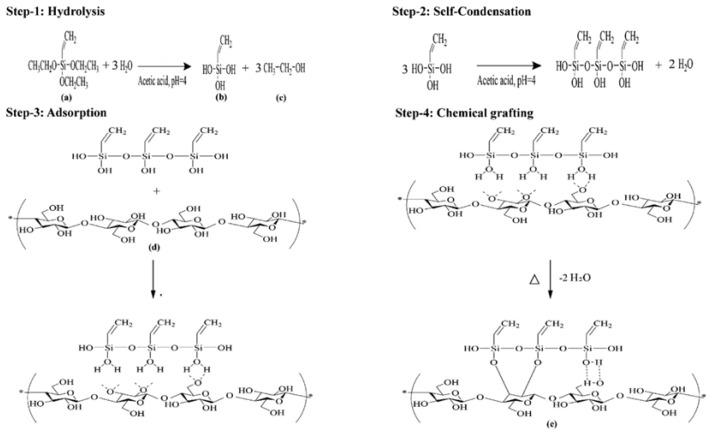
Schematic diagram of CNCs modified by TEVS coupling compound, (**a**) TEVS; (**b**) silanol; (**c**) ethanol; (**d**) cellulose nanocrystals (CNCs); (**e**) modified cellulose nanocrystals (m-CNCs). (TEVS = Triethoxyvinylsilane) [64]. Copyright 2022, Elsevier Ltd.

Dhali et al. [64] adopted a simple and eco-friendly method to partially substitute surface hydroxyl groups by linking polysiloxane, thereby imparting hydrophobic characteristics. In Figure 7, A silanization reaction was conducted, in which triethoxyvinylsilane (TEVS) was hydrolyzed into reactive silanols, followed by condensation to form branched polymers. These polysiloxane oligomers were chemically grafted onto CNC surfaces, forming alkoxysilane bonds. The modified CNCs achieved an appropriate hydrophilic-hydrophobic balance, improving their dispersibility in hydrophobic matrices such as PBAT. Similarly, Thakur et al. [65] investigated the surface functionalization of lignocellulosic Eulaliopsis binate (*E. binate*) fibers through mercerization and silane treatment using vinyltrimethoxysilane as a coupling agent. The effects of silane-induced functionalization on the physicochemical behavior of *E. binate* fibers were subsequently analyzed. The results indicated that vinyltrimethoxysilane-mediated functionalization significantly influenced the physicochemical properties of the fibers. These surface-modified fibers can serve as components for developing green polymer composites.

**Figure 8:**
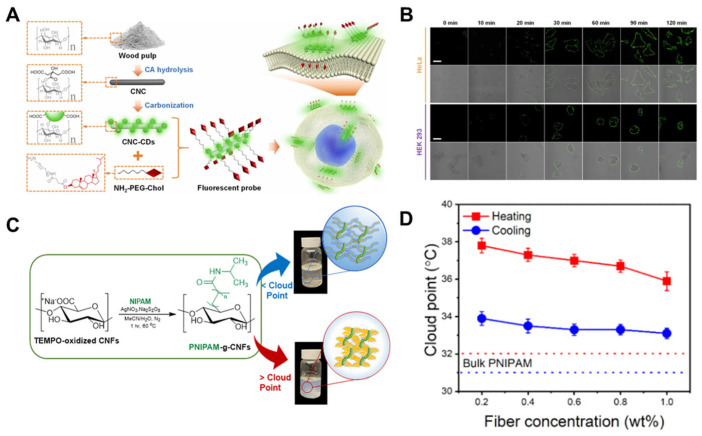
(**A**) Illustration of the construction procedure of the CNC-CDs@PEG-Chol probe and its application in plasma membrane imaging. (**B**) Fluorescent and fluorescent/bright field overlaid images of HeLa cells and HEK 293 cells treated with the CNC-CDs@PEG-Chol probe as a function of time. Scale bar: 20 μm. (**C**) Silver-promoted decarboxylative polymerization of NIPAM from TEMPO-oxidized CNFs. (**D**) Effect of fiber concentration on the cloud point of PNIPAM-g-CNF suspensions. Copyright 2024, Elsevier Ltd. [67]. Copyright 2022, American Chemical Society [68].

The plasma membrane governs various cellular events, including material transport, signal transduction, and pathogen recognition. Fluorescence imaging technology is widely recognized for its inherent high spatiotemporal resolution and its ability to design probes that “illuminate” microscopic targets [66]. Through meticulous probe design, imaging of numerous plasma membrane components or microenvironments has been realized. However, these probes are susceptible to photobleaching under laser irradiation or internalization by live cells, often providing only narrow imaging time windows, which significantly limits their utility in tracking membrane dynamics. To address this, Liu et al. [67] developed a novel fluorescent probe for plasma membrane imaging in Figure 8A. The probe consists of a CNC scaffold extracted from low-cost wood pulp, carbon dots (CDs) synthesized in situ on CNC surfaces, and a cholesterol (Chol) ligand linked via an amino-polyethylene glycol (PEG) spacer. In the CNC-CDs@PEG-Chol configuration, the hydrophobic cholesterol ligand exhibited excellent binding affinity for the plasma membrane, while the CDs provided outstanding optical stability and high quantum yield. The unique morphological properties of the CNC scaffold enable dense assembly of cholesterol ligands and prevent diffusion of imaging molecules into the cytoplasm (Figure 8B). Consequently, this probe demonstrates remarkable specificity and a satisfactory imaging time window (30–90 min of co-incubation), making it a promising tool for studying plasma membrane behavior related to cellular physiological processes.

Mendoza et al. [68] synthesized a series of thermo-responsive PNIPAM-grafted CNFs using a novel Ag(I)-promoted decarboxylative polymerization method (Figure 8C). The approach relied on TEMPO-mediated oxidative decarboxylation of carboxylic acid groups to generate radicals on CNF surfaces. The polymerization was conducted under mild conditions (e.g., aqueous solvent, short reaction time, low temperature, and non-toxic materials) in a single step. Rapid C-C bond formation between CNFs and PNIPAM was achieved, while free polymers were generated in solution. The degree of functionalization (DF) and the amount of grafted PNIPAM could be controlled by adjusting silver concentrations. Similar to bulk PNIPAM, PNIPAM-grafted CNFs (PNIPAM-g-CNFs) exhibited significant thermos-responsive behavior, albeit with slight hysteresis during heating and cooling cycles. Grafting PNIPAM onto hydrophilic CNFs increased the cloud point from approximately 32 °C to 36 °C in Figure 8D. Unlike physical blending, covalent bonding transformed inert CNFs into thermosensitive biomaterials. The cloud point of PNIPAM-g-CNFs remained largely unaffected by silver concentration but slightly decreased with increasing fiber concentration. Rheological studies confirmed sol-gel transitions in PNIPAM-g-CNFs, with storage modulus (G’) above the cloud point increasing with grafted PNIPAM content. This novel chemical method paves the way for polymerizing any vinyl monomer from CNFs or carbohydrate surfaces. The study validates a new strategy for grafting PNIPAM onto CNFs, enabling the synthesis of thermo-responsive transparent hydrogels for diverse applications.

#### 2.2.3. CNC-Hybridized Hydrogels for Biofunctionalization

**Figure 9:**
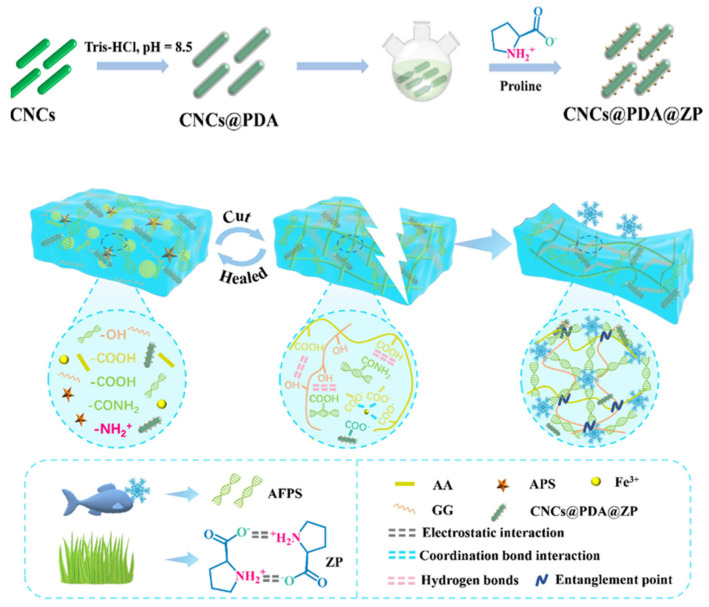
Schematic diagram of the synthesis process and interaction mechanism of hydrogels [69]. Copyright 2025, Elsevier Ltd.

While CNCs can enhance mechanical properties, their tendency towards self-aggregation limits their effectiveness. Consequently, surface modification is required. Modified CNCs can serve both as nano-fillers and as multifunctional cross-linkers. Inspired by mussel-adhesion chemistry, Bai et al. [69] introduced dopamine (DA) onto CNCs to enhance adhesion and provide chemical cross-linking sites. Further modification with oligoproline (ZP) yielded cellulose nanocrystal@polydopamine@oligoproline (CNCs@PDA@ZP), which effectively mitigates self-aggregation. These CNCs@PDA@ZP, along with antifreeze proteins (AFPs), were incorporated into a multicomponent network composed of poly(acrylic acid) (PAA), guar gum (GG), and FeCl_3_, fabricating an antifreeze, self-healing nanocomposite hydrogel flexible sensor. The CNCs@PDA@ZP contributed additional cross-linking sites, hydrogen bonding, and electrostatic interactions. The introduced AFPs exhibited a synergistic effect with the modified CNCs, enabling the hydrogel to achieve a self-healing efficiency of 81.7% within 1.3 h under conditions of 20 °C and a fracture stress of 3.0 MPa. This strategy significantly enhanced the hydrogel’s mechanical properties, antifreeze performance, and sensing sensitivity (Gauge Factor, GF = 4.0). These attributes render the material promising for applications in wearable devices, biomedical sensors, and flexible electronic skin (Figure 9).

**Figure 10:**
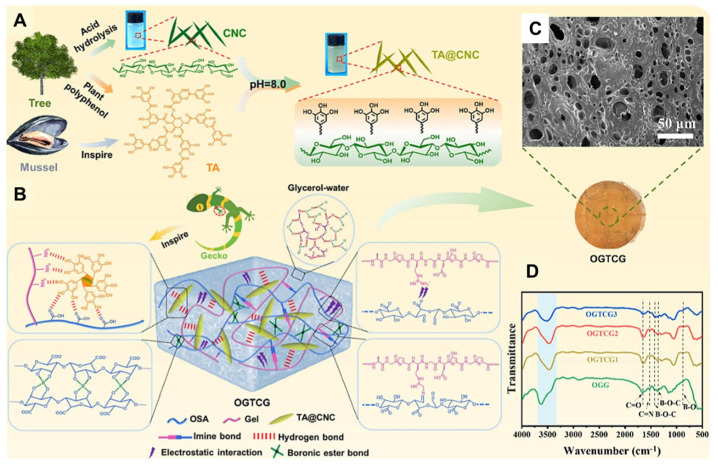
Schematic illustration showing the fabrication of the (**A**) TA@CNC and (**B**) OGTCG hydrogels. (**D**) FT-IR spectra, and (**C**) SEM and real images of the OGTCG hydrogel [71]. Copyright 2024, Elsevier Ltd.

Compared to electronic devices based on metals or semiconductors, hydrogels are promising for wearable applications due to their softness, adaptive tunability, and excellent conductivity. Ion-conductive hydrogels, formed by salts or ionic liquids, are ideal materials for flexible sensors that monitor human health [70]. However, producing hydrogels with high sensitivity, multifunctionality, transparency, and UV-blocking capabilities remains challenging. As shown in Figure 10, Cui et al. [71] addressed this by designing a fully biomass-based hydrogel (OGTCGs) via a one-step method. Tannic acid-coated CNCs (TA@CNCs) were incorporated to block UV light, while oxidized alginate (OSA), gelatin (Gel), TA@CNCs, and a water/glycerol (Gly) binary solvent with borax were combined to prepare the hydrogel. Inspired by gecko fibrillar arrays, hydrogen bonds formed between TA@CNCs and natural polymers in the OGTCG hydrogel. The TA@CNC-reinforced hydrogel exhibited high toughness (218.67 kPa) and modulus (100.32 kPa). Additionally, the hydrogel retained transparency, with TA@CNCs providing UV-blocking effects. Synergistic interactions among amine bonds, borate bonds, and hydrogen bonds endowed the hydrogel with self-healing properties. By adding Gly, the OGTCG hydrogel remained stable for a long time. The OGTCG hydrogel has antibacterial, non-toxic, and high sensitivity in effectively detecting human joint motion (GF = 3.97). The green strategy proposed in this study to develop a multifunctional hydrogel platform can be used for smart wearable devices and has great application potential in monitoring human health.

**Figure 11:**
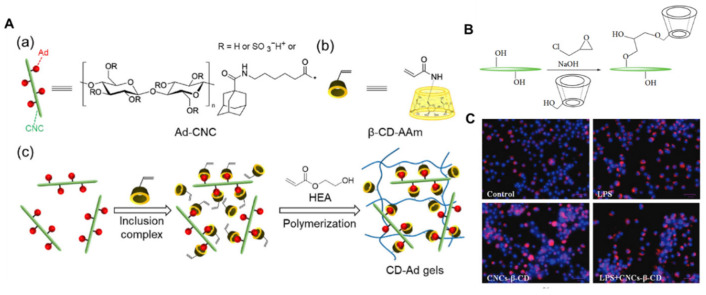
(**A**) Chemical structures of (**a**) the Ad-modified CNC filler (Ad-CNC) and (**b**) the host monomer, 6-acrylamido-β-CD (β-CD-AAm). (**c**) Schematic illustration of the preparation process employed to obtain the CD-Ad gels. (**B**) Reaction scheme for the preparation of CNCs-β-CD. (**C**) Effect of CNCs-β-CD on the changes in mitochondrial ROS in LPS-primed macrophages (scale bar = 40 μm). Copyright 2023, Elsevier BV [72]. Copyright 2019, Wiley-VCH Verlag [73].

In Figure 11A, Myat Noe et al. [72] functionalized CNCs with guest adamantane (Ad) molecules through a simple esterification reaction, leveraging the abundant hydroxyl groups on CNCs’ surfaces. The geometrical dimensions and morphology of the unmodified CNCs were preserved, ensuring dispersibility. The well-dispersed Ad-modified CNCs acted not only as reinforcing fillers in polymer networks but also as supramolecular cross-linkers via host–guest interactions between β-CD and Ad. Supramolecular composite hydrogels with improved strengths were prepared via free radical copolymerization of 2-hydroxyethyl acrylate (HEA) and 6-acrylamido-β-CD (β-CD-AAm) in the presence of Ad-CNCs. The formation of host-guest inclusion complexes on Ad–CNCs surfaces enhanced interfacial compatibility between the filler and polymer matrix. These results therefore confirmed that supramolecular bonding at the filler/matrix interface was responsible for the enhancement of the final mechanical properties of the composite hydrogels.

Sunasee et al. [73] examined the potential immune and antioxidant response induced by CNCs grafted with β-cyclodextrin (CNCs-β-CD) in a human monocyte cell line (THP-1) and a mouse macrophage-like cell line (J774A.1), shown in Figure 11B. They analyzed the secretion of the proinflammatory cytokine, interleukin 1β (IL-1β), by ELISA and mitochondria-derived reactive oxygen species (ROS) using fluorescence cell imaging and examined the intracellular levels of proteins involved in the immune and antioxidant response by immunoblotting. Our results indicated a dramatic increase neither in the IL-1β secretion nor in the mitochondria-derived ROS, resulting in no changes in the intracellular antioxidant response in THP-1 cells treated with different concentrations of CNCs-β-CD. Overall, CNCs-β-CD is nonimmunogenic and does not induce an increased antioxidant response under the conditions tested, and hence has the potential to be used as a drug delivery carrier.

**Figure 12:**
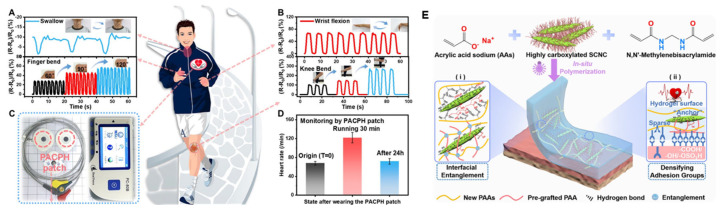
Potential applications of the PACPH patch. The PACPH patch was attached to different locations in the human body to monitor human activity based on the changes in current caused by changes in the activity, such as (**A**) swallowing (top) and bending of the fingers (bottom) and (**B**) bending of the wrists (top) and bending of the knees when walking (bottom). (**C**) The photographic image of a long-lasting ECG-electrode-integrated PACPH patch and the device used to monitor the ECG signal. (**D**) The variation in human heart rate during different activity states during long-term monitoring lasting one day using the PACPH ECG electrode patches. (**E**) Schematic illustration of the fabrication and network structure of the tough, highly adhesive/conductive nanocomposite hydrogel patch (PACP_X_H; X is the content of SCNCPA). (**i**) Schematic illustration of interfacial entanglement; (**ii**) Schematic illustration of densified adhesive groups. Copyright 2024, Royal Society of Chemistry [75].

Adhesive hydrogels (AHs) are considered ideal materials for flexible sensors [74]. However, the lack of an effective energy dissipation network and the scarcity of surface polar groups in hydrogels lead to poor mechanical properties and interfacial adhesion, thus limiting their practical applications. Lu et al. [75] prepared a tough, long-lasting adhesive and highly conductive nanocomposite hydrogel (PACPH) via the synergistic effect of strong interfacial entanglement and adhesive groups densification in Figure 12E. PACPH was obtained by the in situ polymerization of highly carboxylated cellulose nanocrystals (SCNCPA, surface pre-grafted with polyacrylic acid chains, C-COOH = 11.5 mmol g^−1^) with an acrylic acid precursor. The unique tacticity of SCNCPA provides strong interfacial entanglement and multiple hydrogen bonds to the PACPH network, which further increases the energy dissipation during the displacement process of SCNCPA and enhances the PACPH’s mechanical properties (Figure 12A–D). The abundance of polar groups on the surface of SCNCPA solves the problem of the scarcity of polar groups on the surface of hydrogels. As a result, densifying the adhesion groups and high energy dissipation synergistically improved the adhesion properties of PACPH, which demonstrated an adhesion force of 5.5 N cm^−1^ and an adhesion strength of 65 kPa (>2–3 times). The excellent and stable electrical conductivity of PACPH further endows it with more application potential, such as the persistent and stable monitoring of human activities and electrocardiogram signals under different exercise states, which is essential to promote the application of adhesive hydrogels in human health management. For example, the heart rate was stable in the resting state (68 ± 4 min^−1^) and accelerated in the exercising state (122 ± 11 min^−1^). Health management is crucial for promoting the application of bonded hydrogels in human health management for further development of flexible sensors and other health management devices.

**Figure 13:**
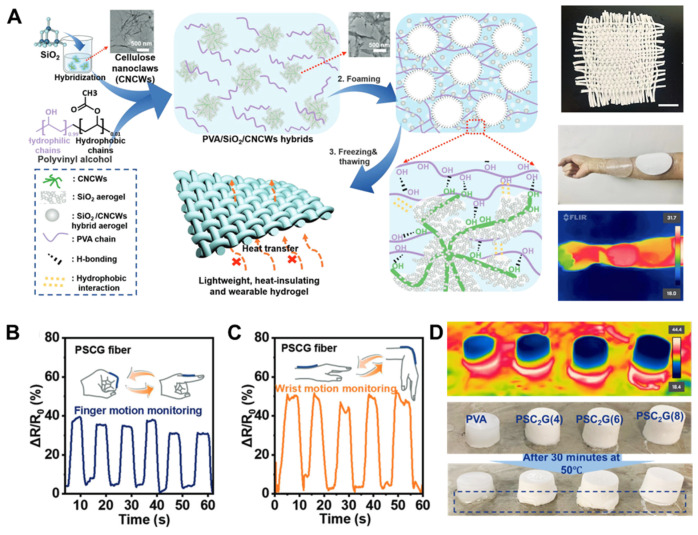
(**A**) Schematic illustration of lightweight and thermal insulation braided hydrogel based on spongy PVA/SiO_2_@CNCWs hydrogels (PSCG). (**B**) The PSCG fibers are assembled as monitors to detect the extent of the bending of a finger or the (**C**) flexing of a wrist. (**D**) Thermal insulation of PVA hydrogels and PSCG [76]. Copyright 2023, Wiley-VCH Verlag.

Li et al. demonstrated an effective hybrid hydrogel–aerogel strategy to assemble ultralight, insulating, and tough PVA/SiO_2_@cellulose nanoclaws (CNCWs) hydrogels (PSCGs) via strong interfacial interactions of hydrogen bonding and hydrophobic interactions, as shown in Figure 13A [76]. The generated PSCGs have interesting hierarchical porous structures derived from bubble templates, PVA hydrogel networks introduced by ice crystals, and hybrid SiO_2_ aerogels, respectively. The PSCGs can be cut into stripes with scissors and then woven into hydrogel fabrics for wearable hydrogel devices, and PSCGs exhibited better heat-insulating performance compared to PVA hydrogel. (Figure 13D) The PSCG is connected to an inductance, capacitance, and resistance (LCR) meter. When the finger is bent, the change in resistance can be sensitively measured (Figure 13B). As shown in Figure 13C, different resistance responses were observed when the wrist was bent in different directions. These findings indicate that the PSCG is capable of accurately monitoring the bending motion of the limb and has further application prospects in the field of wearable soft electronic devices. At the same time, Li et al. [77] also demonstrated a muscle-inspired shape memory-guided PVA–natural rubber latex (NRL) hydrogel (OPNH) with a multiscale oriented structure. Reconfigurable interactions between PVA and NR during stretch drying produced a multilayered oriented structure that improves mechanical and shape memory properties. The strategy of stretching-induced crystallization and orientation endowed OPNH with anisotropic mechanical properties. In addition, the authors simulated the application of OPNH in bionic muscle robotics. This tough, anisotropic, high-performance muscle-inspired hydrogel opens up new avenues for applications in fields such as biomimetic smart devices.

In conclusion, CNC-reinforced hydrogels exhibit exceptional mechanical strength, dimensional stability, toughness, and tunable swelling behavior, making them suitable for tissue engineering, drug delivery systems, wound healing, biosensors, and wearable devices.

## 3. Bioinspired Structural Design and Intelligent Actuation Behavior

By mimicking the hierarchical structure and functional properties of living organisms in nature, the biomimetic structural design of hydrogels significantly improves the mechanical properties, dynamic responsiveness, and environmental adaptability of the materials and has become a core strategy for promoting the development of smart soft materials [78]. In the field of flexible electronics, the ionic conductivity, low interfacial impedance, and mechanical compatibility of hydrogel brakes with human tissues make them an ideal choice for wearable sensing devices and implantable devices [79,80]. For example, strain sensors based on polyvinyl alcohol/polypyrrole composite hydrogels can output electrical signals in real time with the bending of human joints, and at the same time, realize a self-adjusting fit function through humidity response [81]. In the direction of soft body robotics, the flexibility of hydrogel breaks through the traditional rigid actuator on the limitations of freedom of movement, and its imitation of muscle contraction–diastolic behaviors (such as acrylic/clay nanocomposite hydrogel under the electric field of 40% strain) and octopus tentacle-like entanglement grasping ability for medical minimally invasive surgical robots, disaster relief robotic arm provides a new design paradigm [82,83]. In biomedical engineering, the biocompatibility and microenvironmental responsiveness of hydrogels have been further amplified: pH-sensitive gelatin-based hydrogels can be used for controlled release of intestinal-targeted drugs, and temperature-responsive PNIPAM hydrogel scaffolds can promote directional migration of cells through the lysis-contraction cycle, which have all demonstrated their potential in tissue engineering and regenerative medicine [84,85]. Notably, the environmental adaptability of hydrogel brakes is being qualitatively enhanced by bionic structural design. Anisotropic cellulose nanocrystalline reinforced hydrogels inspired by plant cell walls can achieve directional deformation similar to the opening and closing of pinecones through humidity gradients; gradient cross-linked hydrogels mimicking the multilayered structure of the skin integrate tactile sensing and self-repairing, laying a material foundation for the next generation of interactive soft robots.

**Figure 14:**
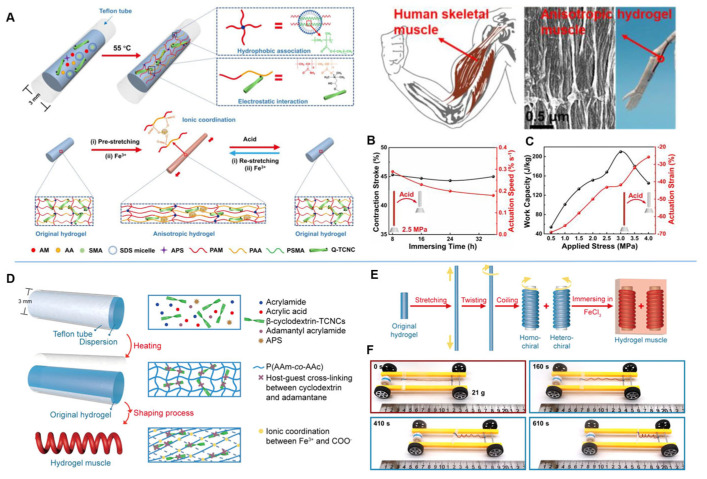
(**A**) Schematic illustration of a dual physically cross-linked hydrogel. (**B**) Contractile stroke and actuation speed of hydrogel muscles with various immersing times under a loading of 2.5 MPa. (**C**) Stress dependence of actuation strain and work capacity of hydrogel muscles [86]. (**D**) Schematic illustration of the fabrication process and network structure of the hydrogel artificial muscle. (**E**) Shaping process for obtaining homo- and heterochiral hydrogel muscles. (**F**) Movement of the car model with the homochiral hydrogel as the motor, driven by water spray [87]. Copyright 2023, Elsevier Ltd. (**A**–**C**). Copyright 2021, American Chemical Society (**D**–**F**).

As shown in the Figure 14A, Chen et al. [86] inspired by the ordered structure of skeletal muscle, through electrostatic interactions between positively charged tunicate cellulose nanocrystals (TCNCs) and -COO^−^ in polymers, hydrophobic association of stearoyl methacrylate (SMA) moiety in sodium dodecyl sulphate (SDS) micelles and -COO^−^/Fe^3+^ ionic coordination, they developed a triple physically cross-linked network consisting of high-performance anisotropic hydrogels. In Figure 13B,C, based on the excellent mechanical properties enhanced by TCNCs, the developed hydrogel artificial muscles exhibit high actuation stroke (75%) and high work capacity (210 J kg^−1^), higher than skeletal muscles. These hydrogel muscles have high stroke, high work capacity, and output efficiency comparable to that of natural muscles, and thus have potential for a wide range of applications. Similarly, in Figure 14D–F, Cui et al. [87] designed a continuous shaping process to fabricate tendril-inspired hydrogel artificial muscles. TCNCs were incorporated into the polymer network through host–guest interactions and were able to enhance the strength of the hydrogel. Dendritic hydrogels can be obtained by treating TCNC-enhanced hydrogels through successive stretching, twisting, and curling processes and locking the shaped structures by Fe^3+^/-COO^−^ ion coordination. These hydrogel muscles have high actuation rates, roughly dynamic strains, and shape memory properties towards solvents. In addition, homochiral hydrogel muscles with temporary shape II show contractile work capacity comparable to that of natural muscles and can be used as an engine to actuate the movement of a car model. This work has great potential for biomedical applications.

**Figure 15:**
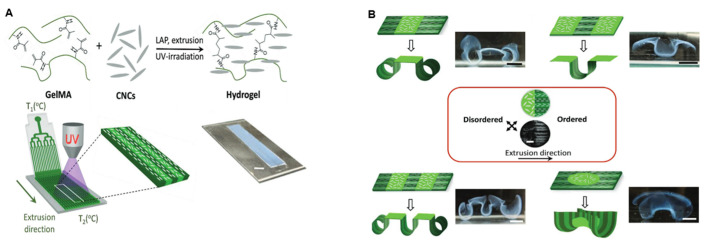
(**A**) Extrusion-based 3D printing of the nanocolloidal CNC/GelMA ink. (**B**) Swelling-induced actuation of CNC/GelMa hydrogel sheets photopatterned with ordered and disordered regions (shown with dark-green and bright-green colors, respectively). The scale bars are 10 mm. Central panel: the scale bar is 2.5 mm. Copyright 2021, Wiley-VCH Verlag [90].

With the introduction of advanced manufacturing technologies such as 3D printing and microfluidic molding, hydrogel actuators are accelerating from laboratory prototypes to practical applications [88,89]. However, further breakthroughs are still needed in long-term stability, dynamic response accuracy, and multi-field coupling control mechanisms, which will be a key challenge for the realization of “material–structure–function” integrated smart systems. For example, Gevorkian et al. [90] reported a 3D-printed single-layer nano-colloidal hydrogel actuator that undergoes a stimulus-triggered shape transition solely due to structural anisotropy. The hydrogel was formed from rod-like CNCs and methacryloyl gelatin (GelMA). The CNC built structural anisotropy through orientational alignment, and during 3D printing, shear forces oriented the rod-like CNCs in the direction of extrusion to form a uniaxially oriented structure. As shown in Figure 15A, the orientation was “locked” in the hydrogel network by physical cross-linking (e.g., hydrogen bonding, electrostatic interactions) and subsequent photo cross-linking. Moreover, the oriented CNCs acted as rigid nanofillers and formed strong interfacial bonds with the GelMA matrix along the orientation, significantly enhancing the longitudinal tensile modulus. The mechanical anisotropy resulted in direction-dependent deformation of the hydrogel when subjected to force, and the dynamic physical interactions (hydrogen bonding, hydrophobic interaction) between CNCs and GelMA gave the ink shear-thinning properties (viscosity decreases by 96–98% with increasing shear rate) to ensure smooth printing, and the viscosity recovers quickly after shear stops (70% recovery rate) to maintain high fidelity and orientation stability of the printed structure. CNCs provided structural guidance and mechanical actuation, GelMA built the dynamic matrix and regulates the rheological properties, and LAP achieved light-controlled cross-linking, all three of which work in tandem to trigger intelligent actuation through structural anisotropy in Figure 15B The mechanism provides a new idea for the development of intelligent soft robots and drug delivery systems that do not require a chemical composition gradient and rely only on structural design.

**Figure 16:**
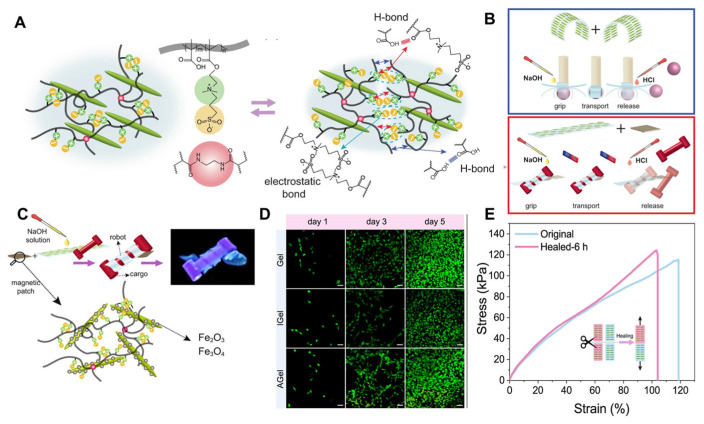
(**A**) Schematic of the self-healing mechanism of the hydrogel through noncovalent reversible cross-linking. (**B**) Schematic of the cut-and-paste strategy to design functional tethered (blue box) and untethered (red box) soft grippers. (**C**) Schematic of a micro-robot transferring a light cargo by twisting around it, triggered by increasing the pH. By adding a magnetic patch to the untethered soft gripper, it can be navigated and steered by an external magnetic field. (**D**) Proliferations of the incubated Fibroblast cells with Gel, IGel, and AGel samples were monitored by fluorescent microscopy over 5 days. Scale bars are 50 μm. (**E**) Self-healing of AGel in perpendicular directions after 6 h [91]. (Copyright 2023, Springer Nature (Berlin, Germany)). Reproduced with permission.

In hydrogel actuators, spatial swelling/shrinking in the aqueous environment in response to an external stimulus manifests in the form of macroscale shape morphing. A well-known method to program shape-morphing is to introduce anisotropy into the hydrogel microstructure to induce differential swelling. Local internal stresses arising from such differential swelling of hydrogels result in anisotropic shape transformations such as twisting, bending, or folding. Nasseriden et al. [91] synthesized a hydrogel composite based on a zwitterionic monomer and asymmetric CNCs, demonstrating broad application prospects in small-scale soft robotics. Researchers designed a synthetic protocol based on the copolymerization of 3-dimethyl(methacryloyloxyethyl)ammonium propanesulfonate (DMAPS) and methacrylic acid (MAA) in the presence of CNC nanoparticles. Within the P(DMAPS-MAA) hydrogel precursor, the shear-induced alignment of CNCs could be utilized to induce structural anisotropy (Figure 16A,B). The self-healing property of P(DMAPS-MAA) enabled the adoption of a “cut-and-paste” strategy to construct complex stimulus-triggered shape-morphing systems. By combining programmable stimulus responsiveness with the cut-and-paste approach, the researchers demonstrated proof-of-concept robotic functionality in the design of both tethered and untethered soft grippers. Further cytotoxicity studies on this hydrogel revealed its high level of cytocompatibility. The design of this hydrogel material and its programming strategy offer new perspectives for the development of tethered and untethered small-scale biomedical soft robots.

**Figure 17:**
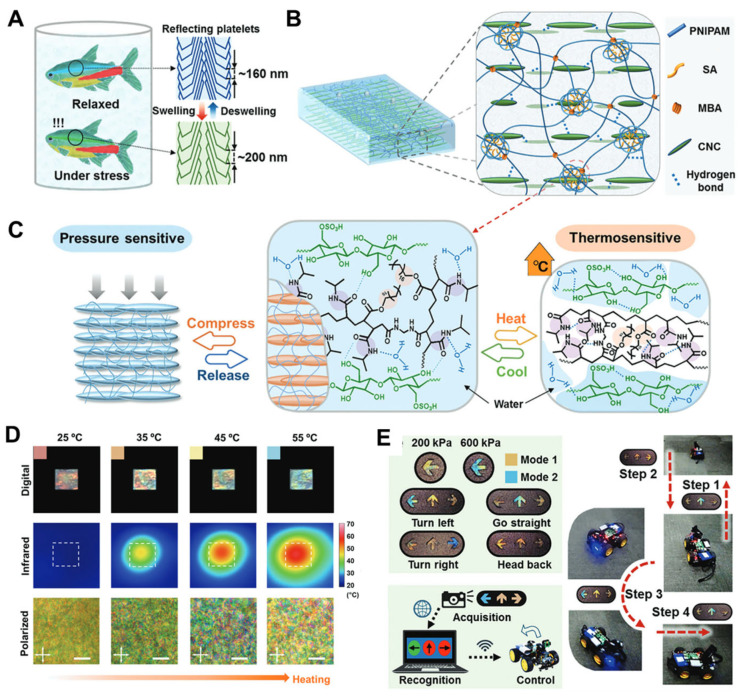
(**A**) The flexible schemochrome of neon tetra based on the adjustable nanostructure. (**B**) Schematic illustration for the biomimetic composite structure of CNC/P(NIPAM-co-PA) hydrogel. (**C**) Scheme of the changing mechanism of dynamic structure colors during the pressure/temperature dual-responsive process. (**D**) Digital photos, infrared thermal images, and polarized optical microscopy (POM) images of the sample during the heating process, scale bar 50 μm. (**E**) Proof-of-concept application of on-demand schemochrome patterning [92]. Copyright 2023 Wiley-VCH Verlag.

Optical devices based on CNCs have garnered significant interest due to their tunable structural colors. However, most previously reported structurally colored CNC-based materials can only achieve simple stress-induced color changes, making it difficult to achieve accurately recognizable multimodal control over complex patterns. Li et al. [92] proposed a novel strategy combining evaporation-induced self-assembly, monomer infiltration and diffusion, and in situ thermal initiation, demonstrating a biomimetic intelligent hydrogel with pressure- and temperature–stimuli-responsive structural colors, as shown in Figure 17A. This hydrogel can flexibly display vivid rainbow structural colors. Leveraging multiple interfacial non-covalent interactions, the researchers incorporated well-aligned CNC cholesteric liquid crystal nanostructures into a thermosensitive copolymer matrix. Based on the temperature-induced volume phase transition of the flexible matrix and nonpolar stearyl groups, the tunable cholesteric helical pitch of the tightly embedded liquid crystal is dynamically correlated with pressure/temperature in Figure 17B. This property endows the resulting hydrogel with dual-stimulus-responsive dynamic structural colors and recognizable visual patterns. The proposed smart hydrogel, featuring dynamically customizable structural color patterns and multi-response control methods, holds promising application prospects in the design of next-generation intelligent optical devices. It is potentially applicable in fields such as smart displays, anti-counterfeiting, temperature monitoring, dual information encryption, and intelligent control systems 

**Figure 18:**
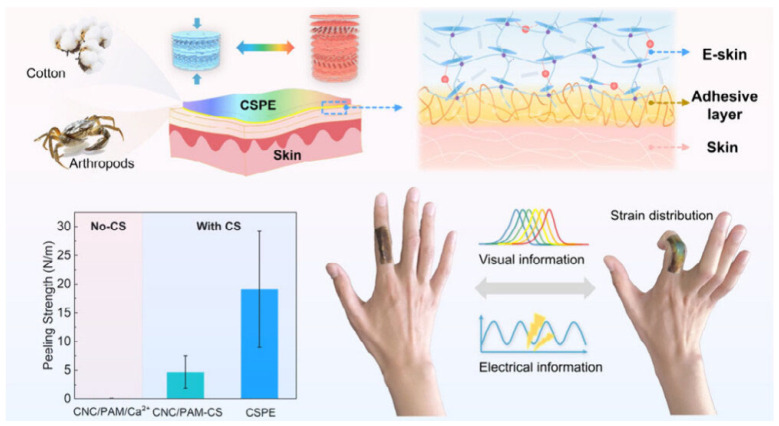
Schematic illustration of cellulose-based, skin-adherent photonic E-skin (CSPE) [93]. Copyright 2025, American Chemical Society.

CNCs exhibit unique color-changing capabilities under external stimuli caused by mechanical strain. Characteristics such as natural origin, cost-effectiveness, biodegradability, biocompatibility, and skin-friendliness make CNC a promising candidate for constructing electronic skins (E-skins). Inspired by the diverse adhesive and responsive structural color phenomena in biological interfaces, Wang et al. [93] developed a cellulose-based, skin-adhesive photonic E-skin (CSPE). This CSPE provides responsive color change and electrical signals under mechanical strain, mimicking the multi-sensory responses of human skin. It has potential applications in health monitoring and sensory prosthetics. As showed in Figure 18, The E-skin design comprises two layers: an upper layer of conductive CNC-based photonic hydrogel for detecting human motion via its reflection peak shift or chromatic change, and a lower chitosan (CS)-based adhesive interface layer ensuring precise tracking through conformal skin contact. The anisotropic adhesion of the CSPE relies on a tough double-network hydrogel as an energy-dissipating matrix and a pH-responsive CS layer that forms a topologically interpenetrating network. This structure enhances adhesive strength by 4.08-fold, achieving a peel strength of 19.12 N/m. They integrated the conductive, tough photonic hydrogel based on CNCs with a layer that bonds the photonic gel to the skin. The photonic hydrogel, with its vibrant structural color, offers quantitative feedback on mechanical stimuli through color mapping and electromechanical changes, enabling precise tracking of human motion. This CSPE technology provides a high-precision, visual solution for glucose-detection tattoos through the innovative fusion of photonic strain mapping and electrochemical sensing. The next steps require focusing on maintaining long-term enzyme activity and shielding against multiple interferents to advance clinical translation.

## 4. Conclusions and Outlook

The biomimetic structural design of cellulose hydrogels significantly improves the mechanical properties, dynamic responsiveness, and environmental adaptability of the materials by mimicking the hierarchical arrangement and functional properties of living organisms in nature, which is a core strategy to promote the development of smart soft materials. Mimicking the orderly arrangement of cellulose in plant cell walls, CNCs are oriented and aligned through shear induction or 3D printing to form a uniaxially oriented structure, effectively overcoming the mechanical shortcomings of traditional hydrogels. By borrowing the multilevel cross-linking network of muscle fibers (e.g., dynamic hydrogen bonding, host–guest supramolecular interactions), hydrogels with sacrificial bonds are designed to achieve multiscale energy dissipation. By multifunctional modification of the CNCs surface, the smart driving behaviors of cellulose composite hydrogels can be precisely regulated. Moreover, conductive-responsive bifunctional hydrogels can be designed by mimicking the multilayered structure of human skin, thus achieving sensing-driving integration.

The hydrogels will focus on the design of multi-scale bionic structures to achieve light–heat–force–electricity multi-field coupled actuation, integrating self-repair, sensing, and actuation functions into a single material system to develop life-like soft robots. In order to accelerate the clinical application, it is particularly important to systematically evaluate the in vivo degradation behaviors and biosafety of bionic hydrogels. In the future, by integrating bionic principles with advanced manufacturing technologies (e.g., 4D printing, microfluidics, and AI-driven design), it will be possible to: (1) Establish a standardized biomimetic evaluation system and develop unified characterization protocols for structure-function correlations in hydrogels (e.g., in situ dynamic mechanical testing platforms); (2) Develop cross-scale modeling tools integrating molecular dynamics (MD) and machine learning (ML) to predict structure–property relationships under multi-field coupling; (3) Advance green manufacturing processes by exploring solvent-free/low-energy consumption forming technologies (e.g., bio-inspired electric field-assisted assembly) to enhance sustainability. Cellulosic hydrogels will lead the next generation of smart materials towards higher complexity, environmental adaptability, and sustainability, and will open up broader prospects for smart materials science.
